# Standardizing test scores for a target population: The LMS method illustrated using language measures from the SCALES project

**DOI:** 10.1371/journal.pone.0213492

**Published:** 2019-03-07

**Authors:** George Vamvakas, Courtenay Frazier Norbury, Silia Vitoratou, Debbie Gooch, Andrew Pickles

**Affiliations:** 1 Department of Biostatistics, Institute of Psychology, Psychiatry and Neuroscience, Kings College London, London, United Kingdom; 2 Psychology and Language Sciences, University College London, London, United Kingdom; 3 Department of Psychology, University of Surrey, Guildford, United Kingdom; University of New Mexico, UNITED STATES

## Abstract

**Background:**

Centile curves and standard scores are common in epidemiological research. However, standardised norms and centile growth curves for language disorder that reflect the entire UK local school population do not exist.

**Methods:**

Scores on six language indices assessing receptive and expressive functioning of children were obtained from the SCALES population survey. Monolingual English speaking participants were aged between five and nine years. Children who attended special schools at study intake, or who were learning English as an additional language were excluded. We constructed language norms using the LMS method of standardisation which allows for skewed measurements. We made use of probability weights that were produced from a two-step logistic model. Distributions of estimated standard scores from an intensively assessed sub-population and from the full population were contrasted to demonstrate the role of weights.

**Results:**

Non-overlapping centile curves and standardised scores at each age were obtained for the six language indices. The use of weights was essential at retrieving the target distribution of the scores. An online calculator that estimates standardised scores for the measures was constructed and made freely available.

**Conclusions:**

The findings highlight the usefulness and flexibility of the LMS method at dealing with the standardisation of linguistic and educational measures that are sufficiently continuous. The paper adds to the existing literature by providing population norms for a number of language tests that were calculated from the same group of individuals.

## Introduction

Developmental cognitive, behavioural and language studies are awash with measures and scales that generate scores that need to be interpreted relative to the child’s chronological age. This process is referred to as test standardisation and involves the positioning of a measurement on a reference age-dependent population distribution, and mapping the measurement to either a centile or a standard score of some kind. In this way an individual’s observed measurement can be compared against expectations based on the distribution or their change over time (velocity) assessed in relation to their progress towards or away from some norm. Sometimes the published standard scores have been derived from a large, carefully selected and fully representative sample of children. Sometimes the tests give the impression of having done this and yet careful reading of the documentation indicates that the low functioning participants, in whom many users of the scales take a special interest, may be poorly represented or largely omitted. It is also not uncommon for a span of low raw scores to be attributed to the same clearly nominal standard score. In almost all cases, the standardisation methodology is not fully described, so that users must simply take on trust the scores provided. Finally, through lack of a suitable sample and the knowledge as to how to do it, many scales simply provide no standard scoring at all.

The most widespread methods for standard scoring generate z-scores (or standard deviation scores), centiles, and centile growth curves. Z-scores refer to the deviation of the measurement from the population mean expressed in units of standard deviation of the reference population. Centiles denote the percentage of people in the reference population the individual’s measurement exceeds or equals. The use of centile curves can be traced back to the 1960s with the development of reference curves of stature and weight for British children [1]. In 1978 WHO/NCHS used least squares cubic splines to construct centile curves for children’s heights, weights, and head circumferences [2]. In 1991 Must, Dallal, and Dietz [3] used the LOWESS method, a non-linear regression technique, to develop growth curves on BMI for people between 6 and 74 years of age. A comprehensive review of different methods that construct growth curves can be found in Borghi et al. [4]. These authors classify the different methods into those that use groupings of adjacent time-points (“bin” methods) and those that treat time continuously, with either class capable of incorporating distributional assumptions about the measurements. In this paper we illustrate one of these methods, namely the LMS method, which treats time continuously and makes some distributional assumptions. This method has gained prominence as the preferred technique in a number of studies, such as the Center for Disease Control and Prevention growth charts [5], the World Health Organisation growth standards [6], and the World Health Organisation growth references [7], due to its efficient use of data and flexibility.

The LMS method was introduced by Cole in 1988 [8] and then refined by Cole and Green in 1992 [9]. It is applied when the measure of interest is strongly dependent on, or changes with time. It allows the data to be analysed continuously on a measure of time and is capable of simultaneous estimation of centile curves at different ages. Estimation of the parameters is done through penalised maximum likelihood and smoothing of the centile curves is achieved through cubic splines. LMS assumes the raw scale scores at a given age are normally distributed after application of a Box-Cox transformation.

In this paper, we develop centile curves and standard scores for tests that assess language disorder in children aged between 5 and 9 years. Our data come from the Surrey Communication and Language in Education Study (SCALES) population cohort [10], which completed screening tests as well as a set of intensive assessments during the first 4 years of formal education (Year 1 and Year 3). We use this sample to construct language norms for the core battery of language tests that were administered to the participants: 1) Expressive vocabulary, 2) Receptive vocabulary (picture identification), 3) Expressive grammar (sentence repetition), 4) Receptive grammar (sentence comprehension), 5) Narrative recall, and 6) Narrative comprehension. Language norms, constructed from the same pool of children, for tasks assessing these specific domains of language have not been developed before, and clinicians have had to resort to standardised values that have been based on disparate samples. To aid clinical practice we develop an online tool that can be accessed free of charge to obtain a child’s standard score on any of the six language indices. To our knowledge, UK population-based standardised norms and centile growth curves for a comprehensive battery of language tests which can be used to identify children with language disorder do not exist. We make extensive use of sampling weights produced by a two-step logistic regression model. The weights help us to account for how the sample of children is different, both by design and non-participation, from the target reference population. This enables us to produce centile curves that reflect the entire mainstream school population and to make sound referenced comparisons of individual standardised scores.

This paper presents the LMS methodology developed for paediatric physical growth data, explains its rationale and illustrates its use for language and behavioural measures. We describe the construction of weights and examine the agreement of the weighted analyses with those from the entire population. Finally, we provide details of the online standard scores calculator and discuss our findings.

## Methods

### Participants and screening

SCALES is a population study on language impairment which involved all state maintained primary schools in Surrey, England. The cohort was selected to be nationally representative using stratified random sampling (see [10] for details). Data were obtained for children who began a reception class in a Surrey school in 2011 (children’s ages ranged from 4 years and 9 months to 5 years and 10 months). The study involved a two-phase design: in the first phase, screening data from 7,267 children were obtained. In this paper, 808 children who attended special schools, or who were learning English as an additional language were excluded. In the second phase, a sub-sample from the remaining 6,459 children was selected for a more detailed clinical assessment to determine whether they had a language impairment; 636 were randomly selected (588 plus 48 children with no phrase speech) for intensive individual assessment at Year 1 and Year 3 of formal education.

Consent procedures and study protocol were developed in consultation with Surrey County Council and approved by the Research Ethics Committee at Royal Holloway, University of London, where the study was initiated. Opt-out consent was adopted for the first phase as data could be provided anonymously to the research team; 20 families opted out. In the second phase, written, informed consent for two episodes of direct assessment was obtained from the parents or legal guardians of all participants. Verbal assent was obtained from children. Prior to assessment in Year 3, families received additional information and the option to withdraw from the study; 18 families withdrew consent, five moved abroad, three could not be contacted and three provided insufficient data on the day of testing for diagnostic classification. Of the 29 children (19 males) not included in follow-up, 22 had been classified as ‘typically developing’ in Year 1 and had no evidence of language, learning or behavioural difficulties. Sixty one percent of all eligible schools took part, and 59% of all eligible children that started school that year provided data. Of the 61% of the schools that took part, 95.38% of children provided data [10].

Screening data from 7,267 children were obtained on the Children’s Communication Checklist-Short (CCC-S) which is a brief version of the CCC-2 [11], and on the Strengths and Difficulties Questionnaire (SDQ: [12]). The CCC-2 has been shown to be highly effective at discriminating children with communication difficulties from typically developing children [13]. The CCC-S contains 13 items from the CCC-2 General Communication Composite that tap language and communication skills in everyday contexts, rated on a 4-point scale. Items measure speech, morphology/syntax, semantics, and discourse skills. Teachers were asked to rate the frequency with which a range of language behaviours occur in everyday contexts on a 4-point scale: 0 = rarely/never, 1 = occasionally, 2 = regularly; 3 = frequently/always. The maximum score of 39 indicates greater disorder. The SDQ is a well-validated questionnaire which elicits teacher ratings of children’s emotions, behaviour, activity levels, peer relationships and pro-social behaviour. There are 25 attributes across the five scales which are rated on a three-point scale as ‘not true’ ‘somewhat true’ and ‘certainly true’. Twenty items from the first four scales (excluding pro-social behaviour) are summed to provide a Total Difficulties Score.

### The intensive sample and test battery

In the second phase, more than 80% of the randomly selected sample took part in the intensive study (see [10] for details). The age range of our participants spans more than four years: children who were assessed in Year 1 of school were aged between 61 and 82 months. Ninety-five percent of them were reassessed in Year 3 and were aged between 85 and 111 months. Data on a range of language indices were collected measuring vocabulary, grammar and narrative skills across two modalities: speaking (expressive language) and understanding (receptive language). These are:

1. *Receptive One-word Picture Vocabulary Test (ROWPVT-4;* [14]). During this test the child hears a word and has to select the corresponding picture, from a choice of four. This task taps semantic memory and draws less on phonological skills than measures of expressive vocabulary. The minimum and maximum scores range from 0 to 190 with higher values indicating a better health outcome. Internal consistency for the age range assessed, 5- to 8-years, as rated by Cronbach’s Coefficient Alpha is 0.95–0.97. Test-retest reliability coefficients for raw scores is 0.97 and for standard scores is 0.91 [14].
2. *Expressive One Word Picture Vocabulary Test (EOWPVT;* [14]*)*. During this test the child is asked to name objects, actions or concepts illustrated in pictures. The score on this task ranges from 0 to 190 with higher values reflecting a better outcome. Internal consistency for the age range assessed, 5- to 8-years, as rated by Cronbach’s Coefficient Alpha is 0.94–0.97. Test-retest reliability coefficients of raw and standard scores are 0.98 and 0.97 respectively [14].
3. *Test of Reception of Grammar–Short Form (TROG-2;* [15]*)*. During this test children hear a sentence such as “the ball that is red is on the pencil” and are asked to select the corresponding picture out of a choice of four. If a child answers incorrectly on six consecutive items, then the test is discontinued. Scores for this task range from 0 to 40, with a higher score denoting more correct responses. As noted in the manual, split-half reliability for the TROG-2 is 0.88 and the resulting correlation suggests good internal consistency (r = 0.877; [11]).
4. *Sentence repetition (SASIT E32;* [16]*)*. During this test the child is asked to repeat 32 sentences out loud. All sentences are pre-recorded and played over headphones to the child one at a time, with a break for the child to repeat the sentence in between. Each repetition is audio recorded and children are scored on whether the sentence is correct or incorrect (minimum score of 0, maximum score of 32), how many of the function words (range: 0–176) and content words (range: 0–121) are repeated correctly and whether the verb (range: 0–44) is correctly or incorrectly inflected. Higher scores on this test indicate more correct responses. The child is given two practice trials to ensure that they understood the task.
5. *Assessment of Comprehension (Narrative Comprehension) and Expression (Narrative Recall) 6–11 (ACE 6–11;* [17]*)*. The child is asked to listen to a story about a monkey in a forest. The story is pre-recorded and played over headphones with accompanying pictures displayed on a laptop computer. After listening to the story, the child is asked to tell the story in their own words (Narrative Recall). The child is given a mark for each part of the story they correctly re-told. The child’s re-telling of the story is audio recorded and the score for this task ranges from 0 to 35; higher scores indicate a better outcome. According to the manual, average internal consistency coefficient (Cronbach’s Alpha) of narrative propositions for children aged 6- to 11-years is 0.73. Assessment of children’s Narrative Comprehension takes place after the Narrative Recall task; the child is asked to answer 12 comprehension questions (6 literal and 6 inference questions) about the story they had just heard. The child is scored 0 for an incorrect answer, 1 point for a partially correct response and 2 points for a correct response. The partially correct and correct responses are determined by a written guide regarding the various possible responses. The score for this task ranges from 0 to 24 with higher scores indicating a better outcome.

These tests were selected to replicate existing epidemiological findings [18] that have informed current diagnostic algorithms for language disorder in the Diagnostic and Statistical Manual of Mental Disorders (DSM5) [19]. Only children who had complete data on the 6 language indices were included in this study (n = 529 for Year 1 (however 1 child did not provide complete data on the day of the assessment); n = 499 for Year 3).

### The LMS method

A thorough description of the method can be found in Cole and Green [9]. The LMS method is based on the Box-Cox transformation [20] that converts the scale raw-score to normality. The acronym LMS comes from the parameters for the Box-Cox power Lambda (L), the median (M), and the generalised coefficient of variation (S). It allows these parameters to vary smoothly with age *t*. The raw scores *y* are transformed to the variable *w* by:

$$w=({\left(y/M\right)}^{L}-1)/L\;,\mathrm{for}\;\mathrm{all}\;L\neq 0\;\mathrm{and}$$

$$w=log_{e}(y/M)\;,\mathrm{for}\;\mathrm{all}\;L=0$$

where M is the median of the untransformed measure *y*. This maps the median of *y* to *w* = 0. Denoting the standard deviation of *w* (or the coefficient of variation (CV) of *y*) by S, then the variable

$$z=({\left(y/M\right)}^{L}-1)/SL\;,\mathrm{for}\;\mathrm{all}\;L\neq 0\;\mathrm{and}$$

$$z=log_{e}(y/M)/S\;,\mathrm{for}\;\mathrm{all}\;L=0$$

is assumed to have a standard normal distribution. Assuming that the distribution of a measure *y* varies with *t*, and that the L, M, and S parameters are read off by smoothed curves L(t), M(t), and S(t) as they vary with age then:

$$z=({\left(y/M(t)\right)}^{L(t)}-1)/S(t)L(t)\;,\mathrm{for}\;\mathrm{all}\;L(t)\neq 0\;\mathrm{and}$$ (1)

$$z=log_{e}(y/M(t))/S(t)\;,\mathrm{for}\;\mathrm{all}\;L(t)=0$$ (2)

Formulae (1) and (2) convert *y* to a skewness-corrected standardised value at each time point. By rearranging (1) and (2) we can derive a centile curve as:

$$C_{a}(t)=M(t){\left(1+L(t)S(t)z_{a}\right)}^{1/L(t)}\;,\mathrm{for}\;\mathrm{all}\;L(t)\neq 0\;\mathrm{or}$$

$$C_{a}(t)=M(t)\exp(S(t)z_{a})\;,\mathrm{for}\;\mathrm{all}\;L(t)=0$$

Where *a* defines the lower tail area of the centile, and z_a_ is the normal equivalent deviate of size a (for example, when a = 0.75 corresponding to the 75^th^ centile, z_a_ = 0.675). Based on (1) and (2), Cole and Green (1992) introduced a penalised likelihood function for the estimation of the parameters that describe the skewness, median, and CV. The degree of the penalty and hence the smoothness of the curves, are controlled by three tuning parameters, referred to as the equivalent degrees of freedom (EDF). There is one EDF for each curve, denoted by a_L_, a_M_, and a_S_, and these three parameters are used to achieve a trade-off between goodness of fit to the data and smoothness of the curves. This latter reduces the risk of too closely following chance fluctuations, called over-fitting, but also reflects a plausible belief that while the domain of measurement may decrease as well as increase and become more or less variable over the population, it rarely develops in erratic jumps.

### Standardisation

Also available as an R-package [21], here the LMSchartmaker software was used for the implementation of the LMS method [22]. LMSchartmaker reads datasets that contain age, a scale score, an optional variable for weights, and an optional ‘grouping’ variable that defines distinct target populations, most commonly the sex of the child in anthropometric studies. In this paper, we have assumed common norms for boys and girls because we do not believe it is acceptable to assume that either sex should have lower expectations of language competence, which is what sex-specific norms would suggest. In addition, epidemiological studies have found no significant sex differences in prevalence estimates for language disorder [10, 18].

As Cole and Green [9] explain, the L(t), M(t), and S(t) curves are constrained to change smoothly with age and how flexibly they can change with age is controlled by the EDFs. The higher the EDF values are, the less smooth the curves appear. Advice on choosing the optimal values for the EDF parameters, and hence the appropriate model for the data, is given in the *User’s Guide to LMSchartmaker*, cited at http://www.healthforallchildren.com/?product=lmschartmaker-pro. We initialised the optimisation process by specifying a_L_ = 0, a_M_ = 1, and a_S_ = 1. A choice of 0 for a_L_ implies no skewness in the distribution of the data at any given time-point, while a choice of 1 assumes that any skewness is constant across the various time-points. Values of a_L_ larger than 1 result in a linear (a_L_ = 2) and non-linear (a_L_>2) change of skewness with age. With regards to the smoothness of the M(t) and S(t) curves, we set the initial values of a_M_ and a_S_ to 1 because zero values are not allowed. A choice of 1 for a_M_ implies no change in the median curve across time. A value of 2 results in a linear increase or decrease, a value of 3 in a quadratic change and anything over 3 in a polynomial M(t) curve. Similarly, for a_S_ = 1 a constant variation is implied; a_S_ = 2 would denote a linear change, a_S_ = 3 a quadratic change, and so forth.

Having chosen the optimal EDFs for L(t), M(t) and S(t), the model was then fitted to the data. For the standardisation of measures at a single time-point, we restricted the values of a_L_, a_M_, and a_S_ to a maximum of 2 in order to prevent over-fitting and achieve more sensible out-of-sample predictions. When we applied the LMS method to data from two assessments points that included an extended age span, we allowed the maximum value of a_L_, a_M_, and a_S_ to be 4.

The manual suggests one should optimise the a_M_ parameter first followed by the a_S_ parameter followed by the a_L_. As a rule of thumb, a_M_ > a_S_ > a_L_. To choose the appropriate model, goodness-of-fit criteria are embedded within the software. These include the Akaike Information Criterion (AIC), Global deviance (G. Deviance), Penalised deviance (P. Deviance), Schwarz Bayesian Criterion (SBC), and the Generalised Akaike Information Criterion (GAIC(3)). Pan and Cole recommend that the most stringent SBC or GAIC(3) deviance criteria should be used. In this paper we focused on values obtained from the SBC criterion and present sequential values of deviance from testing different EDFs.

After the model is chosen and fitted, the software produces several different graphs. These include plots of the original untransformed data against time, the individual L(t), M(t), S(t) curves, centile curves across time superimposed on the raw data, and the Q-test-for-fit goodness of fit plot. While there is no single formal rule as to what is best, particularly with weighted data, we focused on the minimum value of the deviance estimate, obtained by the SBC criterion, on histograms of the standardised values, as well as clinical judgement to inform our choice of model. We required, where appropriate, the deviance value obtained after fitting a model be smaller by more than 2 from the fit of the previous simpler model [23].

The LMS method allows for the construction of any centile curves and we chose to construct curves at the 3^rd^, 10^th^, 25^th^, 50^th^, 75^th^, 90^th^, and 97^th^ percentiles. Weighted and un-weighted densities of the standardised scores were obtained using STATA v.14. While the method ensures that the percentile or standard scores are always consistent across age, in the sense that the 10th percentile will never cross the 5^th^ percentile, over-fitting becomes evident in graphs where the centile curves show implausible variation over age.

### Inverse probability weighting to the reference population

Sampling weights are used extensively in SCALES. They were constructed as the inverse of the predicted probability of a child being included in the study, so that when weighted, the estimates obtained from the sample are estimates for the entire population. Weights are particularly useful because they allow for the oversampling of the lower (or higher) tail of the distribution, where most pathological cases lie but where sampling is often insufficient. In SCALES, children identified by the CCC-S as having a higher risk of a language disorder had a higher probability of being selected to take part in the intensive assessment: a random sub-sample of 636 were drawn from the total of 6,459 children, with a higher sampling fraction for the high‐risk children (40.5% boys, 37.5% girls) versus low‐risk children (4.3% boys, 4.2% girls) and of those, 528 children had complete data on the 6 language indices and were included in this study.

Weights were constructed on a two-step basis, both for the 528 children of Year 1 and for the 499 children of Year 3 who had fully observed data on all language indices. In the first step, to construct weights for children who contributed data at Year 1, a logistic model for whether the child had been invited to participate in the second phase of the study, was fitted to the whole screened sample (n = 6,459). The covariates used were factors predictive of inclusion due to study design, such as whether a child was at high or low risk of language impairment as identified by the screen, and the number of children screened at their school. In the second step, a logistic model was fitted only to children that were selected for the second phase Year 1 assessment. Inclusion also depended on whether children had complete data on all 6 language measures. Since this reflected non-designed participation and response variation, a number of covariates were tested in a stepwise elimination process including gender, income deprivation score, size of school, percentage of girls, special education needs, free school meals, English as additional language, CCC-S score, and number of pupils taking phonics screen. The final weights were constructed as the inverse of the product of the two predicted probabilities. The same two-step process was repeated for the Follow-up assessment in Year 3 resulting in different weights.

In this paper we illustrate the use of LMS method using the CCC-S screening test and the Expressive Vocabulary test. The CCC-S was available on the whole population of 6,549 monolingual children in state-maintained schools. The CCC-S was used solely for the purposes of exploring the role of weights in our analyses, as data on this measure existed on the entire screened sample. This was achieved by visually comparing the estimated distribution of the CCC-S standard scores, obtained from an optimised model fitted to the whole screen population, against the CCC-S standard scores distribution obtained by fitting a weighted and an unweighted model with the same EDFs as the previous model, but to the intensively assessed stratified sub-sample of children only. The dataset of the entire screened sample included child ages and CCC-S raw scores only. This was uploaded into the LMSchartmaker software to initiate the process of model selection for generation of CCC-S standard scores for the entire sample. To create the sub-sample of children, we retained in the dataset participants who provided all six language indices from the intensive study. Children with low CCC-S scores, and thus good language, were markedly under-represented in this sample (by design). This was accounted for by the weights. Hence, the dataset contained values of CCC-S for children who took part in Year 1 only, along with child ages and weights for Year 1. To obtain weighted CCC-S standard scores we fed all three variables into the software and to obtain unweighted standard scores we left the weights out.

Data for the Expressive Vocabulary test existed for both measurement occasions (years 1 and 3) on the intensive sub-sample only. We used data from the Expressive Vocabulary to demonstrate the use of the LMS method when repeated measurements on the same participant existed. To create the dataset for LMSchartmaker, the Expressive vocabulary data for the two time periods were arranged in an ascending order, with values from Year 1 preceding those from Year 3. Hence, the dataset contained values of the Expressive Vocabulary at Year 1 and Year 3, children’s ages at the time of assessment, and corresponding weights for years 1 and 3. In the same way, we obtained standard scores and centile curves for the remaining five language indices, the results being presented in S1 File.

## Results

**Fig 1:**
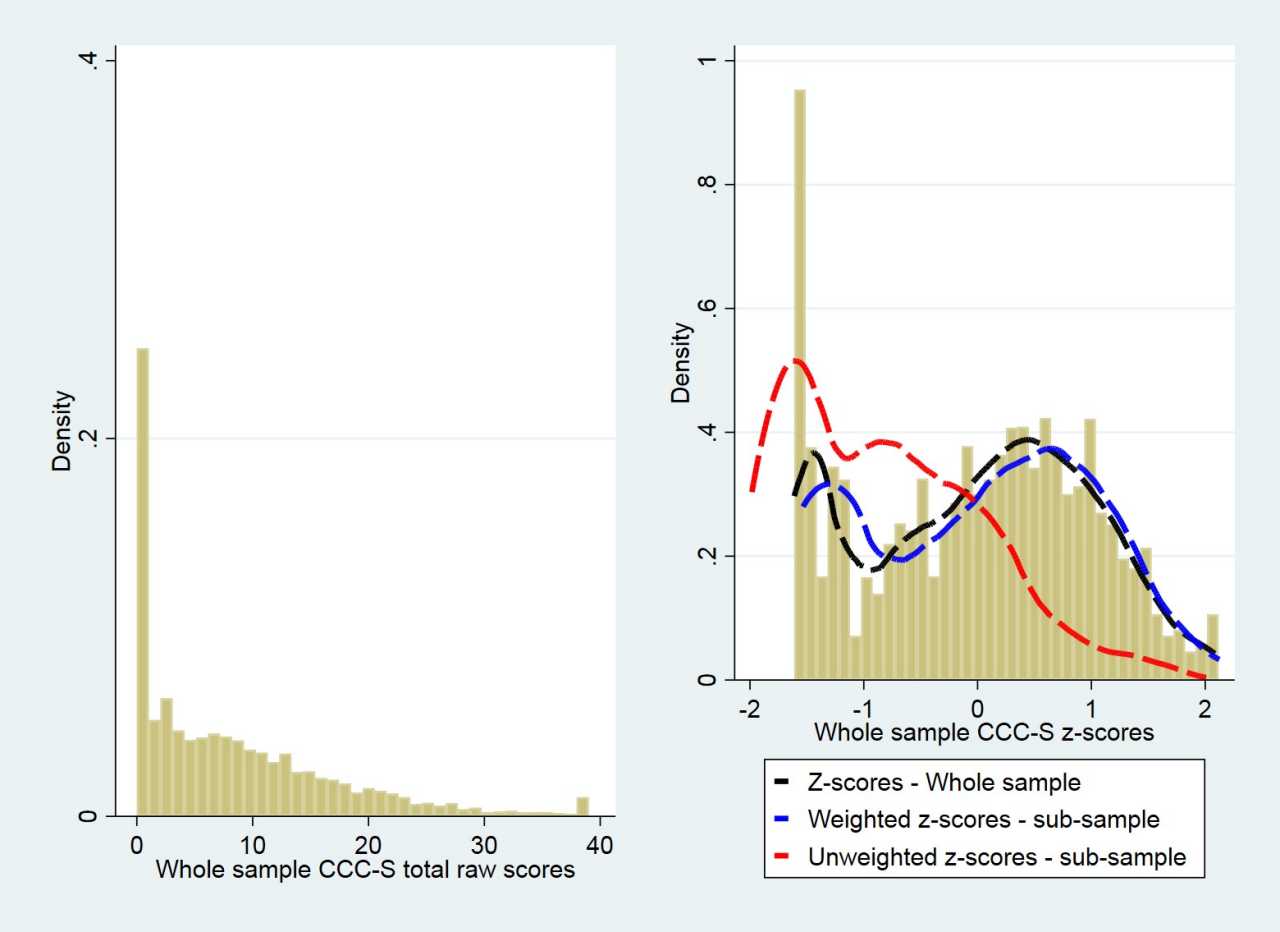
Histograms for raw and standard scores of the CCC-S. Histograms for the raw values (left) and the standard scores (right) of the CCC-S questionnaire, based on the entire sample (n = 6,459). Dashed lines in the right panel depict the Kernel density of the z-scores obtained from the entire sample of 6,459 children (black), and the weighted (blue) and unweighted (red) Kernel densities of the z-scores based on the intensively assessed sample of 528 children.

Total CCC-S scores were available for the entire screened sample (n = 6,459) spanning ages 57 to 70 months. CCC-S total scores range from 0 to 39 with higher scores indicating worse language skills (mean (st. deviation) = 8.5 (8.5), median = 6). As shown on the left panel of Fig 1 the distribution of the raw Total CCC-S values is highly skewed with a mode on zero. The zero mode persisted after standardisation (histogram on the right panel of Fig 1). The skew-minimising Box-Cox transformation requires strictly positive raw-scores so 1 was added to all scores of CCC-S, resulting in a new range from 1 to 40. Table 1 shows the sequential optimisation steps we followed when we increased the EDF values one at a time. It can be seen that we increased the EDF value for M(t) first. When we attempted to increase the EDF value for S(t) next, the model did not converge, so we decided to increase the EDF value for L(t) instead, after which the EDF value for S(t) was successfully incremented. We stopped optimising the model at the highest possible values of EDF at a_L_ = 2/a_M_ = 2/a_S_ = 2 that provided both a reduction in the deviance and a sensible smoothing of the curves. The histogram of the corresponding distribution of the estimated standardised scores produced by this process is shown on the right panel of Fig 1. Details for the dashed lines on the right panel of Fig 1 are presented further below.

**Table 1:** Sequential optimisation process for the CCC-S (n = 6,459).

SBC	Effective degrees of freedom			Difference in SBC
	L	M	S	
45,989.9	0	1	1	-
45,643.9	0	2	1	-346.0
41,821.8	1	2	1	-3822.1
41,819.3	1	2	2	-2.5
41,728.8	2	2	2	-90.5

**Fig 2:**
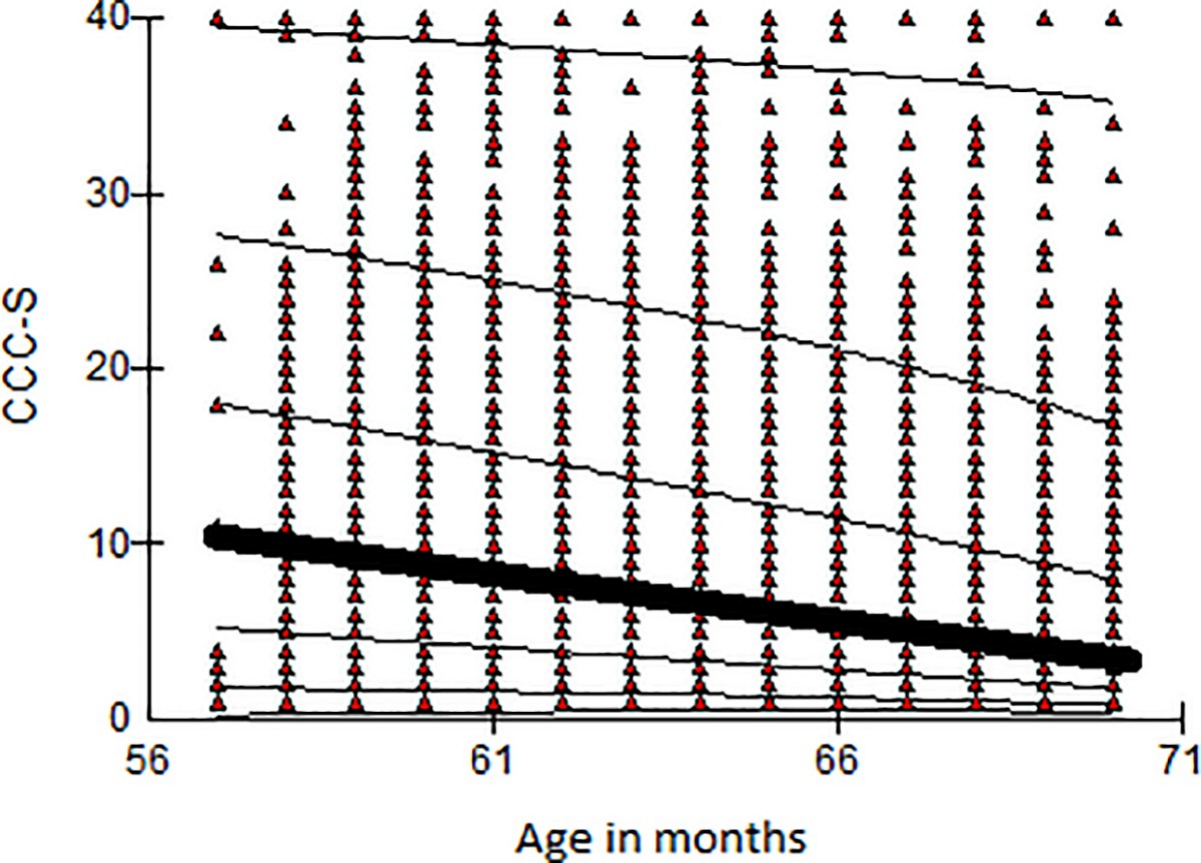
Centile curves for CCC-S. Centile curves for CCC-S based on the entire population (n = 6,459). The bold curve depicts the median centile values across age. The thin lines represent the 3^rd^, 10^th^, 25^th^, 75^th^, 90^th^ and 97^th^ centile curves.

Fig 2 shows the 3^rd^, 10^th^, 25^th^, 50^th^ (median), 75^th^, 90^th^ and 97^th^ centile curves produced by the LMSchartmaker software. The bold line in the middle of the graph depicts the median (50^th^ percentile), which slopes downwards with higher ages, indicating that older children are achieving better CCC-S scores. Since the standard deviation and the skewness of the sample change with age too, the centile curves are not parallel. For example, the curve for the median is steeper than that for the 97^th^ percentile. This allows the expected improvement to depend on the initial scores achieved; the predicted median CCC-S score of a 57-month old child is 11, and the median CCC-S of a 70-month old is 4. Hence, a child who has achieved a score of 11 when they were 57 months old must improve by 7 units by the age of 70 months to maintain their position in the distribution. On the other hand, a child who has achieved a score of 40 when they were 57 months old (top 97^th^ centile curve), must improve by just 4 units by the time they reach the same age of 70 months to retain their position (the 97^th^ centile curve at age 70 corresponds to a CCC-S value of approximately 36).

**Fig 3:**
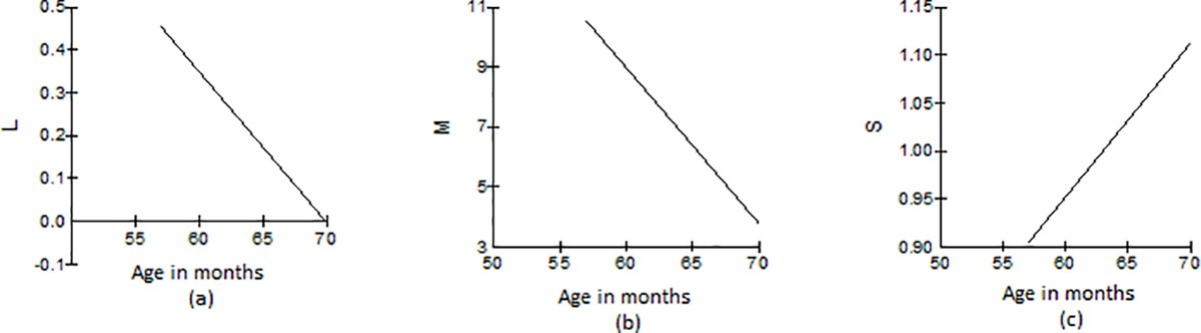
The L,M,S curves. Individual description of how the skewness coefficient (L), median (M), and variation (S) vary with time.

Fig 3 gives a detailed description of how the L(t), M(t), and S(t) curves change with age. We can see the L(t) curve for the skewness coefficient in the standardised CCC-S data is being linearly reduced with higher ages (Fig 3A). The S(t) curve that represents the degree of variation provides an estimate of the variance in the data across age and is linearly increased (Fig 3C). Fig 3B that refers to the median curve, is exactly equivalent to the median curve of Fig 2, and demonstrates the linear decrease for the location parameter M. The L(t) combined with the M(t) and S(t) curves provide all the information necessary to construct standardised scores and centiles.

To illustrate the role of weights in our estimation of standardised scores and centile curves, we created a dataset that contained the ages, CCC-S values, and corresponding sampling weights for the 528 children who provided data at Year 1. We then compared the distributions of z-scores from the weighted a_L_ = 2/ a_M_ = 2/ a_S_ = 2 model and the un-weighted a_L_ = 2/ a_M_ = 2/ a_S_ = 2 model (both based on 528 children) with that from the entire sample of 6,459 children. As the dashed lines on the right panel of Fig 1 show, contrary to the estimated standard score distribution from the unweighted model that was applied to the intensively assessed sample (red line), it is clear that the distribution from the weighted model (blue line) more faithfully resembles the distribution of standard scores obtained from the entire screen sample (black line).

**Fig 4:**
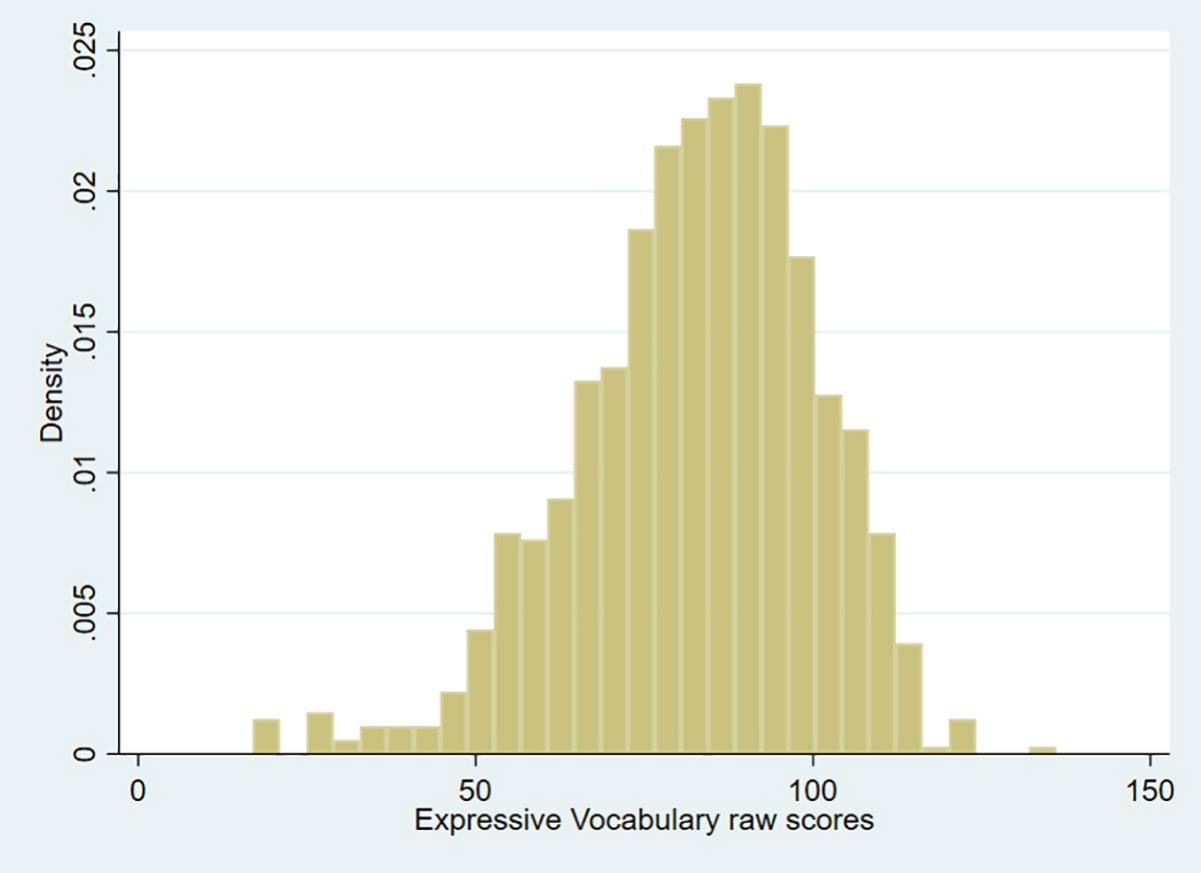
Distribution of the raw scores of the expressive vocabulary test for the combined Year 1 and Year 3 sample (n = 1,029).

Expressive Vocabulary total raw scores were available for 528 children from Year 1, aged 61 to 82 months, and 499 children evaluated at Year 3, aged 85 to 111 months. Table 2 shows descriptive statistics of the raw scores of Expressive Vocabulary before standardisation. Fig 4 displays the distribution of the combined Year 1 and Year 3 raw scores of Expressive Vocabulary.

**Table 2:** Descriptive statistics for raw scores of expressive vocabulary by year.

Expressive Vocabulary	Mean (St. Deviation)	Minimum—Maximum
**Year 1 (n = 528)**	74.5 (15.8)	17, 108
**Year 3 (n = 499)**	91.3 (15.6)	26, 136
**Combined Years (n = 1,027)**	82.7 (17.8)	17, 136

**Fig 5:**
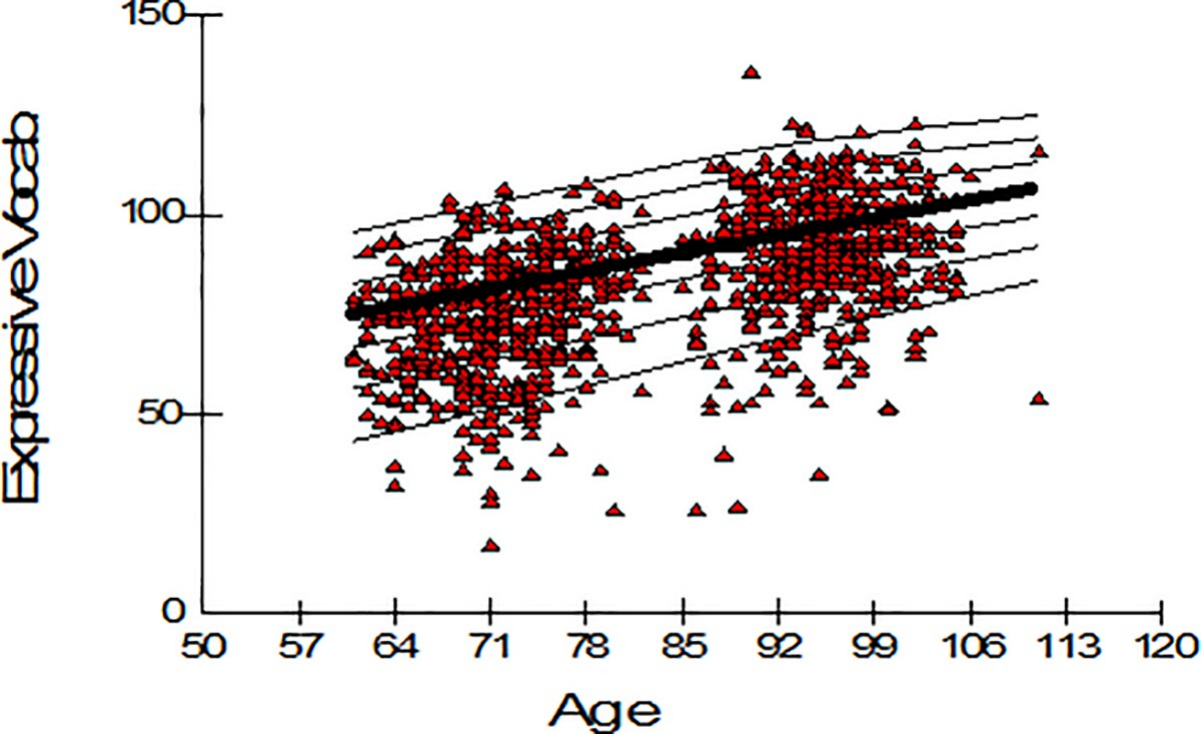
Centile curves for the expressive one word picture vocabulary test. Weighted centile curves for the Expressive One Word Picture Vocabulary test based on the intensively assessed sample at Year 1 and Year 3 (n = 1,027). The bold curve depicts the median centile curve and the thin lines the 3^rd^, 10^th^, 25^th^, 75^th^, 90^th^ and 97^th^ centile curves.

The majority of children had two scores, one from Year 1 and one from Year 3, as well as a set of two different weights for the two time periods. Children who were not seen at Year 3 had one entry only. The optimal model fit was found to be that with EDF values of a_L_ = 1/ a_M_ = 2/ a_S_ = 2 (Table 3), with linear increasing median, linear decreasing variance but age constant skew. Percentile curves are shown in Fig 5. The fact there are more data-points below the 3^rd^ percentile than above the 97^th^ reflects the oversampling of children with poor language, adjusted for during the standardisation process using weights.

**Table 3:** Sequential optimisation process for the expressive one word picture vocabulary test using sampling weights (n = 1,027).

SBC	Effective degrees of freedom			Difference in SBC
	L	M	S	
8330.5	0	1	1	-
7960.0	0	2	1	-370.5
7929.0	0	2	2	-31.0
7869.6	1	2	2	-59.4
7875.5^a^	2	2	2	+5.9

^a^Linear change of skewness increased the deviance so it was kept constant across age.

**Fig 6:**
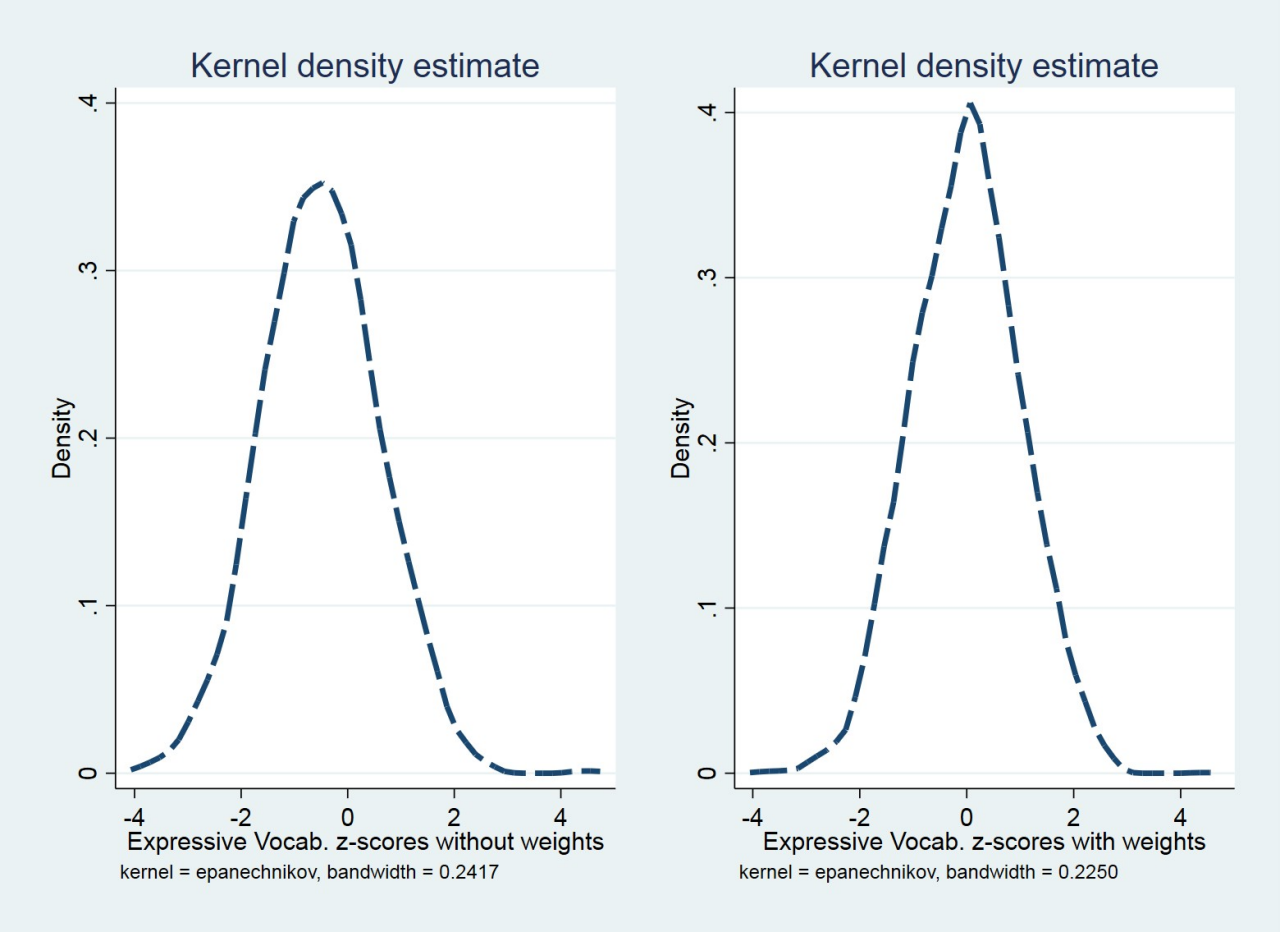
Expressive one word vocabulary standard scores distributions. Distribution of the Expressive One Word Vocabulary standard scores that resulted after the optimisation of the test’s raw scores without weights (left) and with weights (right).

Unlike the CCC-S, which was designed for screening and for which a test floor is not a problem, the Expressive Vocabulary test is intended as a measure able to span the complete ability range. The impact of weights on the optimisation of the Expressive Vocabulary scores is shown in Fig 6. While the centre of the unweighted standard scores distribution falls below 0 (left panel of Fig 6), the weighted distribution of standard scores shown on the right panel of Fig 6 is centred on zero.

### The online calculator

Based on longitudinal information from our intensive sample, and drawing upon the strengths of the LMS method, we have constructed standardised scores and centile curves for all six language indices used in the SCALES study using weights, so that the results are comparable to the entire UK population of mainstream schools. Building upon this, we developed a freely available online calculator where an age-dependent standard score for any of the six language tests, described in the Methods section, can be obtained by simply entering the raw-score and the age of a child. The calculator can be accessed at: https://ioppn.shinyapps.io/SCALES-calculator/.

### Clinical implications

We anticipate that the online tool will facilitate child assessment by supplementing the clinician’s observations with clear, quantified, and comparative information on the child’s language performance relative to peers of the same age. This will aid both diagnosis and prognosis, as well as supporting regular monitoring of language progress. The production of age-dependent standard scores on six language measures can support both speech-language therapists and educators in making targeted assessments of language, identifying expected progress based on current score, and a means of identifying greater than expected progress in response to specific input. In addition, the co-standardisation of measures can facilitate comparisons between the expressive and receptive modalities of vocabulary, grammar, and syntax. Not only does the tool enable targeted monitoring of these aspects of language, it also provides the opportunity for direct comparative assessment of a child’s achievement across the different domains. For example, a clinician might determine a child is performing well at the expressive vocabulary or but relatively poorly on receptive grammar. Intervention therefore might be geared more towards improving receptive grammar. At the same time, it will also be possible to examine how improvement on receptive grammar might influence achievement on other areas of language.

Our population sample included children from across the socio-economic spectrum, though the population of Surrey is skewed towards relative affluence. A number of studies point out that groups of children from lower socio-economic strata have poorer results on language tests, which is true for other aspects of cognitive and behavioural development. Over representation of children with language disorder in areas of socio-economic disadvantage reflects complex interactions between genetic vulnerabilities and environmental circumstances. As we currently lack biological tests for language disorder, it is impossible to know what the underlying contributions to low language performance are, and clinicians should exercise caution in their interpretation of low language scores. No published, standardised tests of language or cognitive ability include separate norms for children from different socio-economic strata and whatever the cause of the language difference, children meeting our criteria for language disorder were substantially below population expectations for age on more than one test of language, and these scores were associated with functional impacts in academic success [10]. Response to appropriate language interventions may further assist in determining whether observed differences from the population mean reflect a cultural difference or a disorder.

## Discussion

We have illustrated, mainly through visual means, the use of the LMS method for generating standardisation charts for two language measures. The non-overlapping centile curves that the method generates is an advantage over more traditional techniques, such as quantile regression.

Our data came from the SCALES population cohort of children who were assessed using a language/communication checklist (at screen) as well as six more intensive assessments of language which are frequently used to identify children with language disorder. This enabled us to analyse the same children using different language indices. We have used the CCC-S measure to demonstrate the role of weights to generate standard scores for a particular target population, and the Expressive Vocabulary measure to show how researchers and clinicians can incorporate repeated measurements in their analyses. The validity of these scores depends firstly upon ensuring that the curves that define the pattern of change in median, variance and skew of the distribution with age have been given sufficient flexibility to match the true underlying variation but not so much flexibility that they are over-fitted (and thus reflect chance idiosyncratic variation of the particular sample data); secondly, that the raw scores are sufficiently continuously distributed that a skew removing transformation to normality is possible, and thirdly that the sample is sufficiently large and representative. Achieving the first of these, while being guided by statistics, is recognised as somewhat of an “art”. Achieving the second and third, depends upon sound age-appropriate test construction, sample design and the potential use of inverse probability weights to account for designed, non-response and post-hoc sampling biases.

At first, we obtained centile curves for the CCC-S questionnaire where values existed for the whole population of 6,549 children aged between 57 and 70 months. As expected, the CCC-S centile curves showed that children improve on average their language ability with higher ages. It was found that the variability of the data as well as the skewness parameter changed in a linear fashion across the ages. Although linearity is not a prerequisite when drawing centile curves, smoothness of the curves is desirable in order to facilitate out-of-sample predictions. We therefore decided, for the given age span, not to exceed a value of 2 in any of the EDF values of the CCC-S measure.

Next, we obtained centile curves and z-scores of CCC-S from the sub-sample of children who had participated in the intensive assessments. This was done to illustrate the effect of weights. Weights were calculated from a two-stage logistic regression to control for the unequal probability of selection and for any systematic non-response in the sample. By contrasting the estimated density distribution of the weighted standard scores with the distribution of the unweighted standard scores, we observed how the weights helped to recover the correct quantities: the centile curves and distribution of the weighted scores from the 528 children, followed those of the original target population of 6,459 children more closely than the centile curves and distribution of the unweighted scores from the 528 children—the latter exhibited a large deviation from the distribution of the target population.

We proceeded to demonstrate the use of LMS in a situation where repeated measurements are taken. According to the methods used in WHO Child Growth Standards (2006), we treated the repeated measurements as if they were generated from a cross-sectional sample where no clustering occurs within the same participant. Data from Year 1 and Year 3 were appended horizontally, and a single set of continuous centile curves was generated across the expanded age span. No more than one measurement from the same child existed within the same age category, and a common set of data from 61 months to 111 months was used to construct centiles and z-scores. We found the optimal model had a constant skewness, while the median and variation changed linearly across age. We showed that the z-scores resulted from the weighted analysis followed the Gaussian distribution better than the distribution of the unweighted z-scores which was skewed to the left. As this sample contained a wider span of ages, the maximum value in any EDF was allowed to be 4. It is possible that researchers may wish to exceed this value when applying the LMS method to their data. For example, Cole and Green [9] demonstrate the use of the method using up to 15 EDFs.

As well as the CCC-S and Expressive Vocabulary measures presented in this paper, we have also produced z-scores and centile curves for the rest of the language indices included in the SCALES study. For our data, the models fell between the simplicity of a_L_ = 0, a_M_ = 1, a_S_ = 1 and the complexity of a_L_ = 2, a_M_ = 3, a_S_ = 3. During this process we found it is often necessary for simpler models to be fitted before the algorithm converges for more complex ones. While the narrative retelling task is part of a larger standardised assessment battery [17] and the full TROG is standardised on a UK population, there are no current UK standard scores for the remaining measures that were included in this paper. In addition, few tests are co-standardised, meaning they are rarely directly comparable even though relative strengths and deficits across language domains is a desirable goal of clinical assessment and intervention planning. We included six robust, evidence-based measures and this paper presents for the first time their standard scores and centile curves based on the same sample of children.

The Supporting Information file contains details of the estimation and optimisation process for the Receptive Vocabulary (Tables A and B and Figure A in S1 File), Receptive Grammar (Tables C and D and Figure B in S1 File), Sentence Repetition (Tables E and F and Figure C in S1 File), Narrative Recall (Tables G and H and Figure D in S1 File), and Narrative Comprehension (Tables I and J and Figure E in S1 File) tests. Standard scores for these measures are available from our online tool which we believe is the first such tool in the field of language.

As Flegal (1999) notes, accurate estimation of percentiles from the LMS method relies on the assumption that after transformation and smoothing, the measure follows the Standard Normal distribution. Although the standardised values of the CCC-S test were not normally distributed, their estimation enabled us to illustrate the effect of weighting. Unfortunately, both screening variables (CCC-S and SDQ) for which information existed on the whole population exhibited a large mode on 0, impossible to fully normalise (details for the SDQ screen are not presented).

The LMS method does not adjust for kurtosis. Although presence of mild kurtosis in the data is not worrying, leptokurtic distributions will result in the wrong extreme centiles. Researchers who encounter significant kurtosis in their data should turn their attention to techniques that adjust for it, such as the method described in Rigby and Stasinopoulos [24].

The popularity of this method is predicated on its ability to model skewed data and construct non-overlapping smoothed centile curves against a measure of time. According to Green and Cole (1992) the advantage of this method is that it fits the three sets of parameters simultaneously making the fitting and the smoothing of the curves an integral part of the maximisation process with less problems of conversion. It saves the researchers from having to make arbitrary choices of age groupings, that older “bin” methods suffered from. As a result, the standard scores and centiles do not depend upon the choice of these age groups, and the sample size becomes less of an issue when constructing the curves [25].

This paper provides a non-technical demonstration of the LMS method on linguistic indices. Although the implementation of the LMS method is widespread in anthropometric disciplines, its usage is not common in linguistic and educational research. We believe, the plethora of sophisticated linguistic and behavioural questionnaires that currently exists necessitates the use of valid and credible practices to produce centile curves and standard scores, and this paper attempts to engage experts with one of them.

## Supporting information

S1 File Vamvakas et al.—PLoS One Supporting Information Appendix. (DOCX)
