# Supplementary material for: Standardizing test scores for a target population: The LMS method illustrated using language measures from the SCALES project
Source: PLoS One. 2019 Mar 7;14(3):e0213492. doi: 10.1371/journal.pone.0213492 (PMC6405128; doi:10.1371/journal.pone.0213492)
Supplement: S1 File — (DOCX) [file pone.0213492.s001.docx]

**S1 PLoS One Supporting Information Appendix**

**Article title:** Standardizing test scores for a target population: The LMS method illustrated using language measures from the SCALES project

**Authors:** George Vamvakas, Courtenay Frazier Norbury, Silia Vitoratou, Debbie Gooch and Andrew Pickles.

The use of the LMS software was found to be straightforward. The tables below display the optimisation sequence in the choice of EDFs necessary to fit the distribution of the raw score adequately, so that the resulting centile curves are smooth and the standard scores follow the Gaussian distribution. Any kind of combination beyond the displayed EDF values would increase the deviance and are therefore not shown. The graphs produced by the LMSchartmaker software, plot the raw scores of the language tests against age in months and display the respective centile curves.

The steps to produce centile curves and standard scores are carefully described in the manual but there were a few instances where we had to deviate from the recommended procedure. The default values of the EDFs are a_L_=3/a_M_=5/a_S_=3. For our analyses we started by optimising the model with a_L_=0/a_M_=1/a_S_=1. The manual states that one should start by optimising the M(t) first, followed by S(t) followed by L(t). This was not always possible to achieve: in some circumstances, revisiting the optimisation of M(t) after having adjusted S(t) or L(t) reduced the deviance of the model further. This was the case for example with Receptive Vocabulary where the combination a_L_=0/a_M_=3/a_S_=1 after having fitted a_L_=0/a_M_=2/a_S_=1 increased the deviance. Further, some combinations of EDF values resulted in non-convergence. The general recommendation of the manual, where a_M_ > a_S_ > a_L_, which as shown in the Tables was not always possible.

Receptive Vocabulary

Table A presents descriptive statistics for the raw Receptive Vocabulary scores before standardisation.

**S1 Table A. Descriptive statistics for the raw scores of Receptive Vocabulary by Year.**

| **Expressive Vocabulary** | **Mean (St. Deviation)** | **Minimum - Maximum** |
| --- | --- | --- |
| **Year 1 (n=528)** | 77.5 (14.1) | 21 , 114 |
| **Year 3 (n=498)** | 97.5 (14.5) | 28 , 139 |
| **Combined Years (n=1,026)** | 87.2 (17.5) | 21 , 139 |

The optimal combination of EDFs for Receptive Vocabulary was found to be a_L_=1/a_M_=3/a_S_=2 (Table B). As such, a quadratic change for the median curve and a linear change for the variation curve were allowed. Skewness was held constant at around 1.75 across the age range. An observation with a very high score (>150) was excluded. This helped us to generate better quality standard scores.

**S1 Table B. Sequential optimisation process for the Receptive Vocabulary test.**

|  | **Receptive Vocabulary** | | |  |
| --- | --- | --- | --- | --- |
| **SBC** | **Effective degrees of freedom** | | | **Difference in SBC** |
|  | **L** | **M** | **S** |  |
| 8411.4 | 0 | 1 | 1 | - |
| 7863.5 | 0 | 2 | 1 | -547.9 |
| 7843.5 | 0 | 2 | 2 | -20.3 |
| 7834.0 | 1 | 2 | 2 | -9.6 |
| 7830.8 | 1 | 3 | 2 | -3.2 |

A scatterplot of the raw values of Receptive Vocabulary against age in months is displayed in Fig A. A dense concentration of values from Year 1 and Year 3 is discernible in the graph: Year 1 values are placed on the left and are generally lower than those from Year 3 encountered on the right. As a result, the centile curves follow a smooth upward direction across the ages. It can be seen that the curves track closely the empirical data; the greater number of observations below the 3^rd^ centile compared to above the 97^th^ centile reflects the oversampling of children with poor language.

**
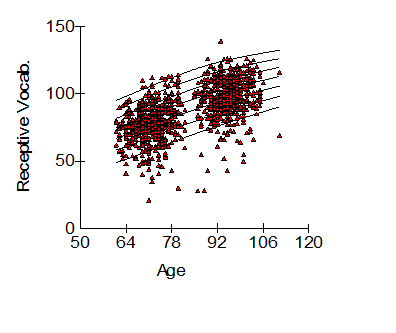
**

**S1 Fig A. Centile curves for Receptive Vocabulary.**

Receptive Grammar

Table C presents descriptive statistics for the raw Receptive Grammar scores before standardisation. The skew-minimising Box-Cox transformation requires strictly positive raw-scores so 1 was added to all raw scores of Receptive Grammar.

**S1 Table C. Descriptive statistics for the raw scores of Receptive Grammar by Year.**

| **Expressive Vocabulary** | **Mean (St. Deviation)** | **Minimum - Maximum** |
| --- | --- | --- |
| **Year 1 (n=528)** | 24.0 (7.8) | 1 , 38 |
| **Year 3 (n=499)** | 30.2 (7.0) | 1 , 40 |
| **Combined Years (n=1,027)** | 27.0 (8.0) | 1 , 40 |

The optimal combination of EDFs for Receptive Grammar was found to be a_L_=1/a_M_=3/a_S_=3 (Table D). Both M(t) and S(t) were allowed to vary quadratically, whereas the skewness was held constant at around 2 across the age span. Although the combination of a_L_=1/a_M_=3/a_S_=2 reduced the deviance by less than 2 points, this was a necessary step to further reduce it by increasing a_S_. This resulted in a more sensible distribution of z-scores.

**S1 Table D. Sequential optimisation process for the Receptive Grammar test.**

|  | **Receptive Grammar** | | |  |
| --- | --- | --- | --- | --- |
| **SBC** | **Effective degrees of freedom** | | | **Difference in SBC** |
|  | **L** | **M** | **S** |  |
| 6349.6 | 0 | 1 | 1 | - |
| 6167.3 | 0 | 2 | 1 | -182.3 |
| 6075.0 | 0 | 2 | 2 | -92.3 |
| 5921.7 | 1 | 2 | 2 | -154.8 |
| 5920.6 | 1 | 3 | 2 | -1.1 |
| 5916.1 | 1 | 3 | 3 | -4.5 |

The scatterplot of the raw data of the Receptive Grammar test is shown in Fig B. The centile curves respect the existence of a greater number of lower scores at Year 1 as opposed to Year 3; lower centiles appear to have a steeper rate of increase during the first Year. Generally, higher centiles appear flatter. All curves are leveling off towards the higher end of the age span.

**
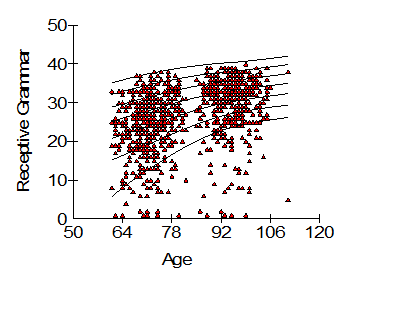
**

**S1 Fig B. Centile curves for Receptive Grammar.**

Sentence Repetition

Table E presents descriptive statistics for the raw Sentence Repetition scores before standardisation. One was added to all raw scores of Sentence Repetition to comply with the Box-Cox transformation requirements.

**S1 Table E. Descriptive statistics for the raw scores of Sentence Repetition by Year.**

| **Expressive Vocabulary** | **Mean (St. Deviation)** | **Minimum - Maximum** |
| --- | --- | --- |
| **Year 1 (n=528)** | 15.2 (8.8) | 1 , 33 |
| **Year 3 (n=499)** | 21.1 (7.9) | 1 , 33 |
| **Combined Years (n=1,027)** | 18.0 (8.9) | 1 , 33 |

The optimal combination of EDFs for Sentence Repetition was found to be a_L_=1/a_M_=2/a_S_=2 (Table F). The median and variation were allowed to vary linearly across age, whereas the skewness was held constant.

**S1 Table F. Sequential optimisation process for the Sentence Repetition.**

|  | **Sentence Repetition** | | |  |
| --- | --- | --- | --- | --- |
| **SBC** | **Effective degrees of freedom** | | | **Difference in SBC** |
|  | **L** | **M** | **S** |  |
| 6231.4 | 0 | 1 | 1 | - |
| 6158.8 | 0 | 2 | 1 | -72.6 |
| 6072.9 | 0 | 2 | 2 | -85.9 |
| 6006.2 | 1 | 2 | 2 | -66.7 |

The centile curves for Sentence Repetition in Fig C exhibit an increasing trend. The initial rate of growth is more rapid for the curves at the bottom of the graph, as these centiles appear to increase at a steeper rate.

**
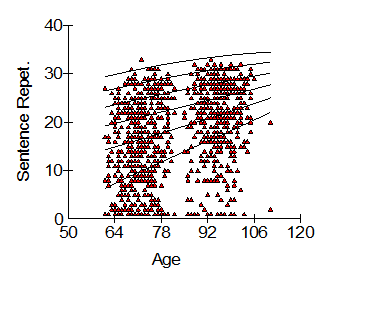
**

**S1 Fig C. Centile curves for Sentence Repetition.**

Narrative Recall

Table G presents descriptive statistics for the raw Narrative Recall scores before standardisation. One was added to all raw scores of Narrative Recall to comply with the Box-Cox transformation requirements.

**S1 Table G. Descriptive statistics for the raw scores of Narrative Recall by Year.**

| **Expressive Vocabulary** | **Mean (St. Deviation)** | **Minimum - Maximum** |
| --- | --- | --- |
| **Year 1 (n=528)** | 11.1 (4.6) | 1 , 24 |
| **Year 3 (n=499)** | 16.2 (4.8) | 2 , 28 |
| **Combined Years (n=1,027)** | 13.6 (5.4) | 1 , 28 |

The optimal combination of EDFs for Narrative Recall was found to be a_L_=1/a_M_=2/a_S_=2 (Table H). The median and variation were allowed to vary linearly across age, whereas the skewness was held constant. Although the a_L_=1/a_M_=3/a_S_=2 combination decreased the deviance, it was decided to choose the a_L_=1/a_M_=2/a_S_=2 model as the difference between the two fits was negligible.

**S1 Table H. Sequential optimisation process for the Narrative Recall test.**

|  | **Narrative Recall** | | |  |
| --- | --- | --- | --- | --- |
| **SBC** | **Effective degrees of freedom** | | | **Difference in SBC** |
|  | **L** | **M** | **S** |  |
| 5901.8 | 0 | 1 | 1 | - |
| 5691.8 | 0 | 2 | 1 | -210.0 |
| 5657.1 | 0 | 2 | 2 | -34.7 |
| 5655.1 | 1 | 2 | 2 | -2.0 |
| 5653.9 | 1 | 3 | 2 | -1.2 |

Again, the centile curves for Narrative Recall in Fig D follow an increasing direction tracking closely the empirical data from Year 1 and Year 3. The spread of the raw datapoints appear to be roughly the same for Year 1 and Year 3. There appears to be a similar change in the rate of increase across all curves.


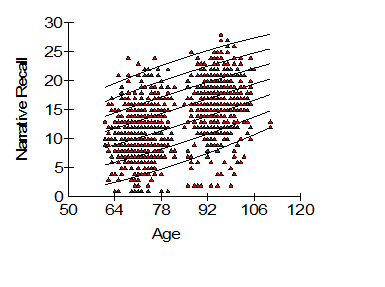


**S1 Fig D. Centile curves for Narrative Recall.**

Narrative Comprehension

Table I presents descriptive statistics for the raw Narrative Comprehension scores before standardisation. One was added to all raw scores of Narrative Comprehension to comply with the Box-Cox transformation requirements.

**S1 Table I. Descriptive statistics for the raw scores of Narrative Comprehension by Year.**

| **Expressive Vocabulary** | **Mean (St. Deviation)** | **Minimum - Maximum** |
| --- | --- | --- |
| **Year 1 (n=528)** | 12.6 (5.0) | 1 , 25 |
| **Year 3 (n=499)** | 16.5 (4.6) | 2 , 25 |
| **Combined Years (n=1,027)** | 14.5 (5.2) | 1 , 25 |

The optimal combination of EDFs for Narrative Comprehension was found to be a_L_=1/a_M_=2/a_S_=2 (Table J). The median and variation were allowed to vary linearly across age, whereas the skewness was held constant.

**S1 Table J. Sequential optimisation process for the Narrative Comprehension test.**

|  | **Narrative Comprehension** | | |  |
| --- | --- | --- | --- | --- |
| **SBC** | **Effective degrees of freedom** | | | **Difference in SBC** |
|  | **L** | **M** | **S** |  |
| 5495.0 | 0 | 1 | 1 | - |
| 5367.3 | 0 | 2 | 1 | -127.7 |
| 5275.2 | 0 | 2 | 2 | -92.1 |
| 5240.1 | 1 | 2 | 2 | -35.1 |

The distribution of the centile curves for Narrative Comprehension in Fig E suggest there is a greater variation among the observations from Year 1 than Year 3 with the curves following a steeper rate of change for the lower centiles.

**
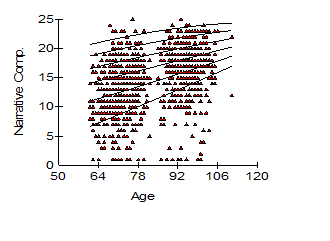
**

**S1 Fig E. Centile curves for Narrative Comprehension.**

Fig F displays the distribution of the raw data of the 5 language indices from the combined Year 1 and Year 3 sample. Fig G shows the weighted distributions of their z-scores resulted from the LMS method. To retain the range of scores between around -4 and 4 standard deviations for Receptive Vocabulary we had to exclude an extreme observation of >150. It appears that the impact of this observation was to cause an exceedingly skewed sample to the left. The shape of the distribution of Receptive Grammar and Sentence Repetition exhibits a slight deviation from normality. This resulted from the fact that during optimisation we were unable to adjust the skewness of the data adequately: any attempt to increase a_L_ would result in non-convergence. Despite this, the shape of the distribution was deemed acceptable given the non-strictly continuous nature of our data.


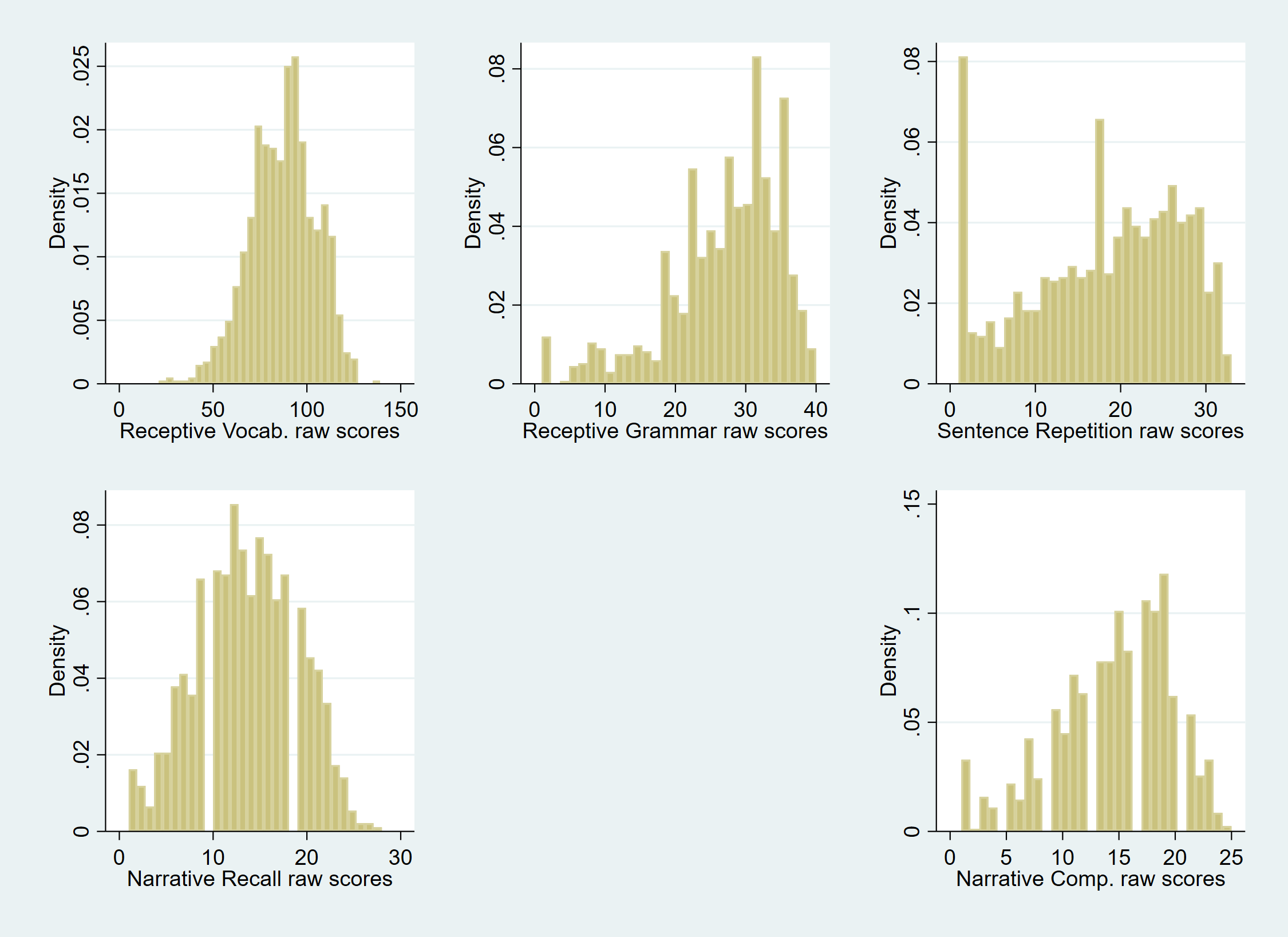


**S1 Fig F. Distribution of the tests’ raw scores from the combined Year 1 and Year 3 sample.**


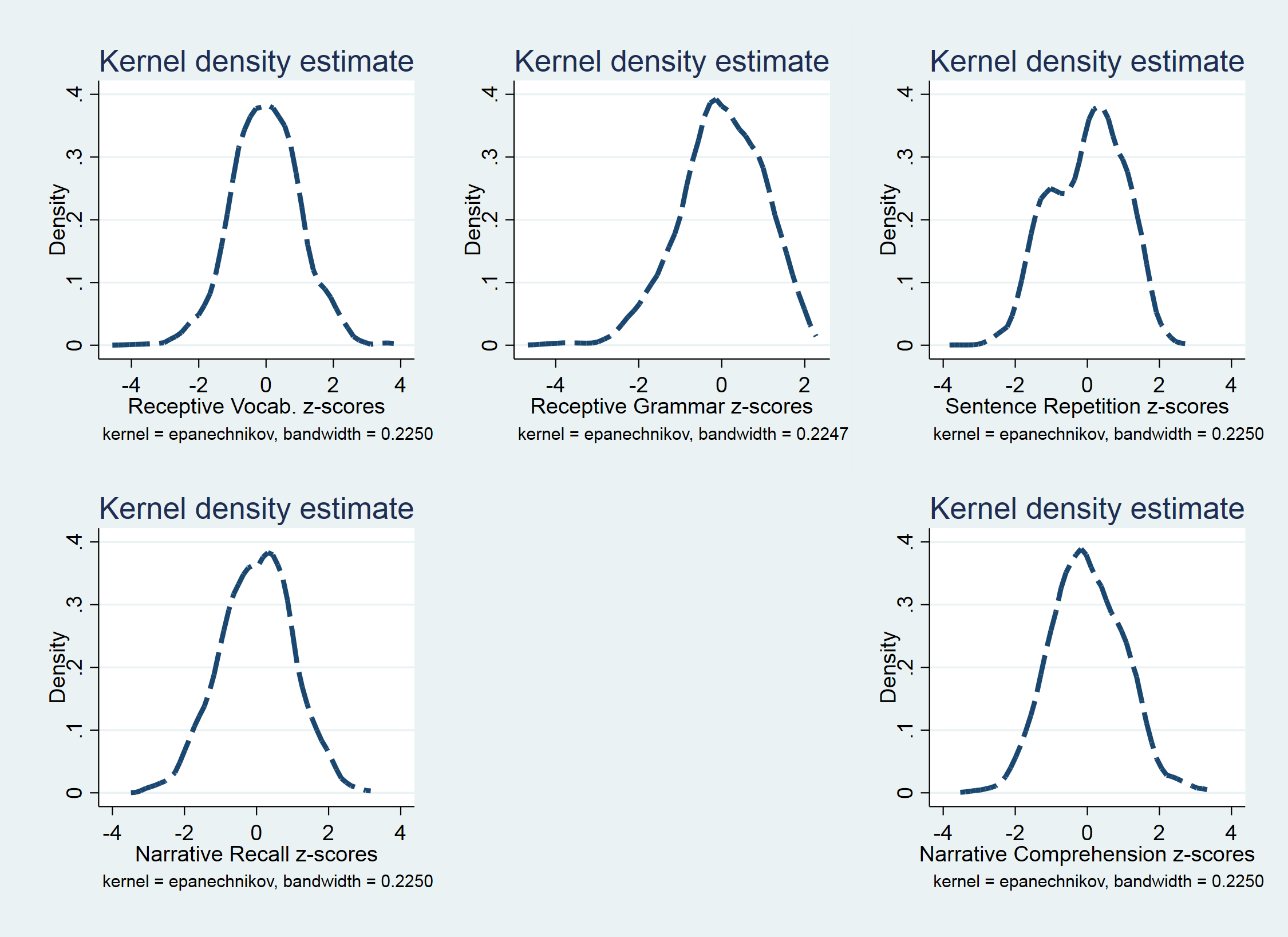


**S1 Fig G. Weighted distribution of the tests’ standard scores.**
